# Current conservative approaches and novelties on pressure sores in patients needing neurorehabilitation

**Published:** 2012

**Authors:** C Popescu, G Onose

**Affiliations:** * The Clinic Division of Physical and Rehabilitation Medicine, “Bagdasar-Arseni” Emergency Hospital, Bucharest, Romania; ** “Carol Davila” University of Medicine and Pharmacy (UMFCD), Bucharest, Romania

**Keywords:** pressure sores, neuromotor impairments, prevention, conservative treatment, physical therapy

## Abstract

**Background**: One major objective in medical units specialized in caring for patients with severe neurological lesions is to reduce the incidence of pressure sores.

**Objective:** A purpose of this article is to give solutions regarding the way to decrease the incidence or progression of pressure ulcer development and offer recommendations for appropriate treatment of pressure sore(s).

**Material and methods:** A systematic literature search was performed based on a practical perspective, a comparative study with two components was conducted: a retrospective and a prospective one regarding the efficiency of a set of prophylactic and therapeutic measures, concomitantly with an enlarged treatment options panel and accessibility to the patient referred.

**Discussion**: It should be pointed out that one characteristic of pressure sores – supplementary enhancing their poor prognostic – is their tendency to recurrence, often meaning augmentation of their severity no matter the therapeutic endeavors approached.

Therefore, prevention remains the key point in medicine and in particular for pressure sores: saves the patient from unnecessary suffering, requires less time and expenses allocated from the budget department.

**Conclusion**: Patients needing neurorehabilitation associate immobility at high risk for the development of decubitus ulcers. The most efficient way to prevent and treat pressure sores, is to early asses and identify their general and respectively specific risk factors in each patient and consequently to promptly initiate prevention or curative appropriate measures.

## Background

Although an ancient medical problem (found in autopsies including on Egyptian mummies) pressure ulcers still affect patients even up to the present day [**1**].

The last decade has accumulated a number of achievements in some sub domains, including the emergency medicine, which led to a somewhat paradoxical result in continuous and marked increase in the number of people - after surviving life-threatening conditions - with different disabilities.

Also, it is marked by the undeniable improvement in patients care, along with their multidisciplinary approach in specialized units, with interdisciplinary teams and by the application of individualized rehabilitation programs, which are sequential, adapted to the neurological, psychological and dysfunctional status of them, prophylactic and therapeutic approaches leading, at least in terms of opportunities, to the participation, to a quasi-normal life or as close to the meaning of that term [**2**].

Thus, the patient needing neurorehabilitation with neuromotor impairments should always be the focus of extensive medical, social and economic measures to prevent both late medical complications and psychological, socio-professional, family and/or economic effects.

Pressure sores are a serious complication of multimorbidity associated with prolonged duration of poor mobility possibilities and in vicious circle worsen the functional outcomes.

After a short introduction to the general problem of pressure sores, the importance of pursuing such issues will have a result; because, with less than a decade ago, and in our country, less than two years ago, there has been a significant discrepancy between the overall prospects of medicine to save lives in patients with severe nevraxial injuries and the relatively weak progress made in both the prevention and control of such complications.

The longitudinal analysis, extended retroactively for a period of 10 years, performed in a representative center in Australia has revealed that pressure sores contribute more than 50% of total readmissions, being incriminated in a disproportionately high number of hospitalization days (on average 65.9 days) [**3**].

The pressure sore is defined as an area of localized damage to the skin and underlying tissue caused by pressure, shearing forces, friction, and moisture [**4, 5**].

Pressure ulcers (also known as bedsores, pressure sores or decubitus ulcers) are caused by compression due to bony projections from inside and respectively the external surface of contact (bed, wheelchair) for a longer – although not always excessive - period. The pressure externally applied on prominent body surfaces, of more than 32 mm Hg, is the primary factor that exceeds the capillary pressure within the tissue, traumatizing the underlying blood vessels, which leads to thrombosis [**6**], with interruption of circulation, hypoxic tissue lesion and finally, necrosis. The critical duration of ischemia that can cause pressure injury varies greatly among individuals; generally, it lies somewhere between 30 and 240 minutes [**7**]; so, changing the position at the right time prevents pressure sore formation. Moisture, friction, poor nutrition, age, low arteriolar pressure are the secondary factors with an important role in the development of bedsores [**8**].

When the tissues are compromised by pressure sore formation, a vascular and cellular response to injury happens. This inflammatory response is the precursor to wound healing and repair. The elevated temperature and leukocytosis are the systemic indicators of inflammation [**9**].

Risk factors for the development of decubitus ulcers should be assessed at the time of the physician’s first contact with an immobile patient.

Generally, the patients needing neurorehabilitation at high risk for pressure sores are those with paralyses and sensitive severe impairments, entailing long periods of mobility limitations such as after: spinal cord injuries, traumatic brain injuries or other severe nevraxial non-traumatic lesions (tumors, infections, ischemic and/or hemorrhagic stokes, hydrocephalia, etc. – neurosurgically approached or non-approached) [**10**].

Additionally, in patients belonging to the afore mentioned pathological conditions, supplementary risk factors are represented by: bladder incontinence (moisture can irritate the skin and thus promote bedsores), low proteinemia (secondary malnutrition - can lead to skin damage and delays in healing), old age (while getting older, the skin becomes rigid and cannot distribute pressure equally; also, the skin becomes thinner and loses its elasticity and thus it is easier to infringe), other associated conditions/ diseases that lower the body's resistance and healing ability, such as hypotension, dehydration, heart failure, diabetes mellitus [**10**].

The prevalence of pressure ulcers in health care facilities seems to be increasing. It is estimated that in hospitalized patients the acute care is 15% [**8**]. In the nursing home environment, the prevalence of pressure ulcers is in the range of 2,6-24% [**9**].

Data reported that pressure ulcer incidence varies from 0,4% to 38% in acute state, from 2,2% to 23, 9% in long term care and from 0% to 17% in home care [**11**].

Another group of experts estimated an overall prevalence of 9.2% among institutionalized patients, based on estimated local prevalence of 5% to 10% in hospitals, about 30% in geriatric clinics and homes for the elderly, and about 20% in nursing-dependent patients being cared for at home [**7**].

The prevalence of high-grade decubitus ulcers (grades 3 and 4) is as high as 3%. It reaches 4% in elderly persons receiving nursing care in institutions [**7**].

Pressure sores are most commonly seen at the sacrum (30 - 60%), ischium (6%), trochanters (6%), heel (30%) [**12**]. Immobility can also cause bedsores on the neck, ear, in the back of the shoulders, the elbows, between the knees, above ankles, in malleolar regions.

Decubitus ulcers are classified by the depth of ulceration and by their site, extent, and wound condition [**5**].

Stage 1: area of intact skin, flushed, persistent erythema, pressing the finger does not change color

**Fig. 1:**
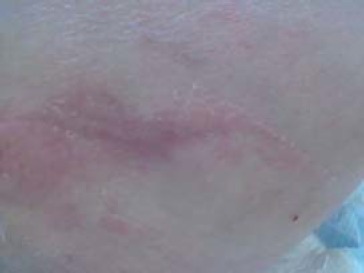
Trochanteric pressure sore - stage 1 (from the casuistry of the Neuromuscular Clinic Division)

Clinical signs: overheating skin, edema (**Fig. 1**)

Stage 2: partial loss of skin layer (epidermis) with damage over the next layer (dermis)

**Fig. 2:**
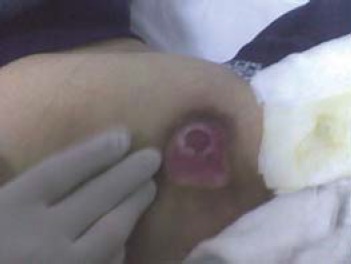
Trochanteric pressure sore - stage 2 (from the casuistry of the Neuromuscular Clinic Division)

Clinical signs: superficial ulcer, with pink-red background, no crust/ exudates (**Fig. 2**)

**Fig. 3:**
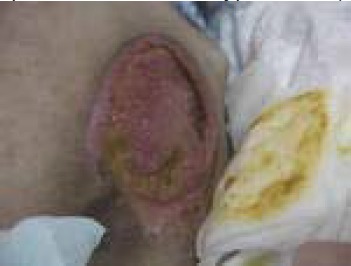
Sacral pressure sore - stage 3 (from the casuistry of the Neuromuscular Clinic Division)

Stage 3: damage to all skin layers (epidermis, dermis, hypodermis), which can reach up to the fascia (**Fig. 3**)

Clinical signs: deep ulceration, with or without damaging the neighboring tissue.

Stage 4: all the layers of skin are damaged and overcome the muscle fascia, impaired the tendons and bones and can be extended by forming pockets bedsores and fistulas.

**Fig. 4:**
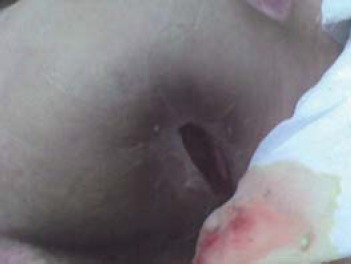
Ischial pressure sore - stage 4 (from the casuistry of the Neuromuscular Clinic Division)

Clinical signs: crust and/or exudate may be present and often eroded fistula - whose depth varies according to anatomical location (**Fig. 4**)

Once a lesion forms, it may progress through four stages marked by worsening necrosis, tissue loss, infection that can lead to sepsis, thus can be life threatening [**13**].

“Spontaneous” healing can take 2-3-4 months; costs thousands of dollars, requiring ointments - for stage 1 -, special dressings (adapted to the evolutionary stage), and infusion of aminoacids derived blood - albumin, plasma, factor XIII, sophisticated physiotherapy equipment. Severe cases require reconstructive surgery, anesthesia or related expense default.

Prevention remains the key point in medicine and in particular for pressure sores: saves the patient from unnecessary suffering, requires less effort, time and expenses allocated from the budget department. Whatever the cause of restraint, from the beginning, complex measures must be taken to prevent bedsores, whether the patient is in hospital or at home.

Two major goals should be pursued in preventing pressure sores: [**14**]

1. Careful skin surveillance

2. Avoiding gravitation pressure on the dystrophic skin exposed regions

1. Careful skin surveillance implies:

a) meticulous skin inspection

Any area of redness, irritation or abrasion is an injury prior to a decubitus ulcer. If the erythematous macule does not fade in 30 minutes, the patient should avoid any pressure in that area until the redness and possible underlying induration disappear. It is prohibited that the patient is positioned on that mentioned side. If within 15 minutes after the removal of pressure, the blurred area does not disappear, further consequences will occur. If after 30-60 minutes after removing the pressure, the erythematous area remains, this is a warning sign that indicates the formation of bedsores (Stage 1).

b) skin palpation - verifying the existence of classic signs of inflammation: rubor, calor and tumor and aside, the possible induration sensation perceived by the clinician

c) body and skin hygiene - skin should be permanently kept dry and clean. Combination like local moisture and heat, friction, forces and/or pressure respectively should be avoided.

d) protection of the skin against repeated lesions that occur during transfer (supported or performed by the patient) – as much as possible

2. Avoiding gravitation pressure on the dystrophic skin exposed regions: correct settlement of the patient, turning regular in bed and/or respectively periodically release of pressure on the buttocks region (ischiatic and partially sacral areas). It is recommended to change position in bed every 2 hours if the bed is not equipped with an anti-bedsores mattress and at every 4-6 hours, if such an equipment is available. If one or more pressure ulcers already exist, the gravitational support on the respective side of the body has to be avoided as much as possible, i.e. when lying in bed, this should be shortened at durations of tens of minutes; if the patient is wheelchair addicted, the appearance of pressure sore(s) on the ischial and/or retro-trochanteric areas, optimally – from the skin healing point of view – sitting in a wheelchair should be avoided unless the ulcer is cured, except for grade 2, in patients having a very modern kind of cushion with electromechanically assisted pulsed airflow. According to our expertise, no other kind of cushions (expanded polymers, with air-cells, filled with gel or at least sponge) can provide a really safe/ effective assistance in this respect, i.e. to permit the patients’ gravitational upload on a pressure ulcer area, whereas this would heal [**2**].

A proper treatment of bedsores scheme should include the following steps:

1. Medical history and general evaluation of the patient’s condition(s), including local ulcer(s) assessment: this implies number, positioning, size, depth, stage of development, color and odor presence of debris, scabs, bacterial colonization, fistulas/ pockets

2. Mobilization - releasing all the pressure on the affected area to restore blood flow

3. Local treatment based on the degree of tissue damage with debridement, modern occlusive hydroactive dressings (based on hydrocolloids, hydrogel, alginate, transparent film, etc.) and sometimes surgery - depending on the developmental stage of the bedsores [**14,15**] and local wound care that eliminates necrotic tissue, decreases bacterial load with general beneficial reverberation upon the overall patient’s stress resistance and tissue recovery capacities, and provides a physiologic, pressure-free environment allowing the wound to heal.

4. The general treatment is prescribed by the doctor for the existing diseases, improvement of nutrition and adequate pain control. It will be tailored to the patient's general status (neurological deficits extension, outreach opportunities, coexisting/ pre-existing pathology, state of nutrition, the patient's possibilities of feeding per os). Assessment of nutritional status in these patients is mandatory and of course the correction of the existing deficiencies, particularly those with multiple pressure sores, large, grade III and/or IV; feeding (per os, enteral and/or parenteral) should provide at least 35 kcal/kgc/day with a protein intake of 1.25 to 1.5 g/kg/day - for people with normal renal function. The treatment plan should include vitamin C and zinc supplements as indicated, systemic antibiotics for sepsis, cellulitis, osteomyelitis or thrombosis prevention with anticoagulant [**14,16**].

**Current and future therapeutic aspects in bedsores, with appropriate details to the evolutionary stage**

Wound dressings are a central component of the pressure sore care. For each of the four stages there are devoted modern dressings, selected according to the tissue aspect.

Hydroactive treatment of pressure sores using modern dressings is possible in stages I, II and III.

Stage IV, with damage to muscles and bones, is an indication for treatment with bandages only after appropriate surgery (deep cleaning bedsores). At this stage (IV), dressings prepare the wound for closure by plastic reconstruction.

Treatment with hydroactive modern dressings consist of autolytic debridement (non-invasive cleaning of the wound with preservation of healthy tissue intact), stimulating the formation of granulation tissue (new cells) and stimulate epithelization (wound closure).

These dressings create the necessary conditions to overcome the stagnant state of the bedsore, due to imbalances at the microcell level, supporting the healing process in each of its stages of development (phase I - cleaning/ exudative, phase II - granulation and phase III - the epithelium).

The plastic surgery to repair the loss of substance is a indication in patients with pressure sores of level III and/or IV, with no healing tendency of conservative treatment, or if the patient wishes a more rapid closure of the ulcer [**5**].

**Topical Treatments [5,17]**

- hydrocolloid dressing, absorptive, for clean stage II pressure ulcers, on non-infected, shallow stage III pressure sores, may be left in place up to several days.

- wound fillers dressing beneath hydrocolloid dressings in deep ulcers to fill in dead space, used on partial and shallow full-thickness minimal to moderate exudate, necrotic and infected.

- hydrogel dressings, available in pad forms (on shallow, minimally exudating pressure sores, for treatment of dry ulcer beds, for pressure sores without depth and contours) and amorphous (for pressure sores with depth and contours, that are not infected and are granulating)

- transparent film dressings: protect body areas for friction injury, promote autolysis, may be used as a secondary dressing for ulcers treated with alginates or other wound filler that will likely remain in the ulcer bed for an extended period of time (3-5 days)

- alginates or other fiber gelling dressings for the treatment of moderately and heavily exudation ulcers, in infected pressure sores

- foam dressings on exudative cavity ulcers stage 2 and shallow stage 3, absorptive, non-adherent to a moist wound bed

- gauze dressings for exudative ulcers, to reduce the evaporation when the tissue interface layer is moist

- collagen matrix dressings for non-healing stage 3 and 4 of pressure sores, stimulates wound healing

- silver-impregnated dressings for pressure sores that are infected or heavily colonized

- contact layers (silicone dressings) protect the wound base, prevent tissue injury when the ulcer or periwound tissue is friable

- enzyme debriding agents, facilitate debridement of necrotic tissue

**Means of physical therapy in the prophylaxis and treatment of pressure sores**

• Electrostimulation of gluteal muscles should improve trophic and vascular tone in the area, aiming to maintain a natural “interface support”, with a protective/ prophylactic role, for redistribution-loaded compared with bone areas - predominantly ischiatic
• Application – paralombar for peripheral circulatory reflex sympathetic modulation - low frequency pulse galvanic current, high voltage - preventively (i.e. for blood flow/ irrigation enhancement in sacral and gluteal regions)
• Direct electrical stimulation of the wound by low frequency currents and intensity, monophasic or biphasic, with electrodes placed near the lesion would accelerate the healing in stages III-IV. Electrostimulation properly applied and with the right intensity is able to affect pressure sores by increasing arterial or nutrient rich blood flow to the affected area, increasing venous or nutrient poor blood flow away from the affected area, increasing the lymphatic flow.
  It is important to mention that such electrostimulation in transcutaneous applications or implanted electrodes were studied, especially in the last two decades but could not, so far, demonstrate effective, in their use to prevent or treat pressure sores [**5,14,18**].
• Studies have been done in connection with possible therapeutic effects in bedsores: low frequency pulsed magnetic fields (only one randomized controlled study with a small number of cases - thus, insufficiently relevant), phototherapy (LASER, infrared, ultraviolet - except polarized light: studies on dozens of cases with favorable conclusions in stages I-III [**19**], ultrasonic therapy, oxygen therapy (topical and/or systemic - hyperbaric) and negative pressure therapy (NPWT - Negative pressure wound therapy) - which seems to improve local blood flow, stimulate granulation and diminish the colonization of the sore [**20**]. Negative pressure wound therapy devices apply negative pressure or suction, to the wound bed and creates three main physical effects: 1) tension on tissues that stimulate mitotic division, 2) increased local blood flow in the capillary bed, and 3) evacuation of excess interstitial fluids. The subsequent therapeutic effects to the wound bed are exudate control, increased granulation tissue formation, reduced wound and periwound edema, increased wound contraction and increased epithelialization [**17,21**].

There is generally insufficient reliable evidence to draw conclusions about the contribution of LASER therapy, therapeutic ultrasound, electrotherapy and electromagnetic therapy to chronic wound healing [**22**].

In addition, the boundary between present and further validation in the future is nanotechnology; the places it has a tendency of occurring being still in clinical testing, of dressings with silver nanocrystals (the antiseptic effect of silver is much known) [**23**].

## Methods

From a practical perspective, the basic design of a comparative study to be developed within the Doctoral Dissertation is herein presented with two components: a retrospective and respectively, a prospective one, regarding the efficiency of a set of prophylactic and conservative therapeutic measures concomitantly with increased treatment options and accessibility to the patient referred.

Regarding the primary prophylactic dimension, a main endpoint considered for the retrospective study is the assessment of bedsores frequency among patients having antisore air mattresses versus the patients who did not benefit from such a device.

Thus, for the prospective study, it is intended, based on informed consents, to obtain the agreement in writing from the enrolled participants and with the Ethical Commission’s approval, to compare the outcomes obtained with electrotherapy (low frequency currents) versus phototherapy (LASER), as physiatric adjuncts to the quite equivalent local therapy based on absorbent, including with antiinfectious properties dressings and generally supportive therapy where needed, currently applied in our Clinic Division.

In this respect, for the prospective component, a unitary assessment protocol comprising the following primary endpoints will be used:

- the length of hospitalization influenced by the presence/ occurrence of bedsores

- frequency of readmissions to the same case due to bedsores (directly or indirectly)

- frequency of cases that have unsuccessfully passed the conservative stage reaching surgery

As for the secondary endpoints, herein below, we present the following parameters: name - encrypted/encoded, gender (F, M), age, diagnosis, general condition, complications (yes/ no), number of bedsores, evolutionary stage, location, dimensions (length, width, depth – in mm), particular/ specific aspects of the lesioned tissue (yes/ no), smell (yes/ no), the time from onset of bedsores (weeks), treatment applied, evolution and also the patient's subjective status rated on a scale with five grades: very negative, bad, balanced on the edge, satisfactory, positive/ optimistic. It is important to mention that the afore-presented five degrees scale will quantify the patients’ general condition, too.

**Discussion and conclusions**

In the treatment of pressure sores, one should take into account the risk of relapse on long term. Studies approaching (also) this subject matter, reported varying relapse rates, between 25 and 80% [**24**] specifically, different other studies report the following values: 48,9% in cases of ischiatic pressure sores surgically treated, respectively a relapse rate of 20,8% in sacral pressure sores [**25**]; other studies observed a relapse in 60% of surgically treated pressure sores with a 82% relapse rate in patients paraplegics [**26**].

Pressure sores are very prompt to recurrence; therefore, aside from the continuous search for the improvement of the therapeutic options both primary and secondary prophylactic endeavors remain a major importance and effectiveness.

The emergence and development of bedsores is influenced by a multitude of factors that transform the patient care in a long process that requires attention.

Alongside the topical treatment of bedsores, it requires continuous pressure reduction using repositioning techniques, skin care, cleaning and protection products and adequate nutrition including general support that was previously described.

One of the methods which do not require surgical intervention and which has proved to be fully effective in the topical treatment of bedsores is based on the use of modern dressings in accordance with their evolution, possibly augmented in efficiency by physiatric approaches, entailing novel ways of therapeutic energy vectors delivery to the affected skin and beneath soft tissues in pressure ulcers.

## References

[1] Idowu OK, Yinusa W, Gbadegesin SA, Adebule GT (2011). Risk factors for pressure ulceration in a resource constrained spinal injury service. Spinal Cord.

[2] Onose G (2007). Rehabilitation, Physical Medicine and Balneoclimatology - basic and current concepts.

[3] Middleton JW, Lim K, Taylor L, Soden R, Rutkowski S (2004). Patterns of morbidity and rehospitalisation following spinal cord injury. Spinal Cord.

[4] Longe RL (1986). Current concepts in clinical therapeutics: pressure sores. Clin Pharm.

[5] European Pressure Ulcer Advisory Panel and National Pressure Ulcer Advisory Panel (2009). http://www.epuap.org/.

[6] Vohra RK, McCollum CN (1994). Pressure sores. PubMed.

[7] Anders J, Heinemann A, Leffmann C, Leutenegger M, Pröfener F, von Renteln-Kruse W (2010). Decubitus Ulcers: Pathophysiology and Primary Prevention. Deutsches Arzteblatt International.

[8] National Pressure Ulcer Advisory Panel (2001). an Executive Summary of the Pressure ulcers in America: prevalence, incidence and implications for the future. Adv Skin Wound Care.

[9] Salcido R, Lorenzo TC Pressure Ulcers and Wound Care. Medscape.

[10] Anghelescu A, Anghelescu C (2001). Elemente de fiziopatologie si neurofarmacologie in traumatismul vertebromedular acut. Medicina Modernă.

[11] Layder CH (2003). Pressure ulcer prevention and management. Jama.

[12] Meehan M (1994). National pressure ulcer prevalence survey. Adv Wound Care.

[13] Grigorean VT (2009). Patologia secundară traumatismelor vertebromedulare.

[14] Onose Gelu, Guidelines on diagnosis, treatment and rehabilitation after suffering spinal cord injury (2011).

[15] Perez ED (1993). Pressure ulcers: updated guidelines for treatment and prevention. Geriatrics.

[16] Goode PS, Allman RM (1989). The prevention and management of pressure ulcers. Med Clin North Am.

[17] Institute for Clinical Systems Improvement - Health Care Protocol (2010). Pressure Ulcer Prevention and Treatment Protocol. http://www.icsi.org/pressure_ulcer_treatment_protocol__review_and_comment_/pressure_ulcer_treatment__protocol__.html.

[18] Regan M, Teasell RW, Keast D, Aubut JL, Foulon BL, Mehta S, Eng JJ, Teasell RW, Miller WC, Wolfe DL, Townson AF, Hsieh JTC, Connolly SJ, Mehta S, Sakakibara BM (2010). Pressure Ulcers Following Spinal Cord Injury. Spinal Cord Injury Rehabilitation Evidence.

[19] Durović A, Marić D, Brdareski Z, Jevtić M, Durdević S (2008). The effects of polarized light therapy in pressure ulcer healing. Vojnosanit Pregl.

[20] Kairinos N, Hudson D, Solomons M (2009). Depth of penetration of negative pressure wound therapy into underlying tissues. Wound Repair Regen.

[21] Gupta S, Cho T (2004). A literature review of negative pressure wound therapy. *Ostomy Wound Manag*.

[22] Cullum N, Nelson EA, Flemming K, Sheldon T (2001). Systematic reviews of wound care management: (5) beds; (6) compression; (7) laser therapy, therapeutic ultrasound, electrotherapy and electromagnetic therapy. Health Technol Assess.

[23] Gavriliu S, Lungu M, Gavriliu L, Grigore F, Groza C (2009). Antimicrobial Colloidal Suspension of Silver-Titania. The Open Chemical and Biomedical methods Journal.

[24] Nae S, Antohi N, Stingu C, Stan V, Parasca S (2010). Cover flaps options in pressure sores. Surgery.

[25] Yamamoto Y, Tsursumida A, Murazumi M, Sugihara T (1997). Long-term outcome of pressure sores treated with flap coverage. Plast Reconstr Surg.

[26] Evans GR, Dufresne CR, Manson PN (1994). Surgical correction of pressure ulcers in an urban center: Is it efficacious?. Adv Wound Care.

